# Sleep-Stage Correlates of Hippocampal Electroencephalogram in Primates

**DOI:** 10.1371/journal.pone.0082994

**Published:** 2013-12-30

**Authors:** Ryoi Tamura, Hiroshi Nishida, Satoshi Eifuku, Hiroaki Fushiki, Yukio Watanabe, Kumiko Uchiyama

**Affiliations:** 1 Department of Integrative Neuroscience, Graduate School of Medicine and Pharmaceutical Sciences, University of Toyama, Toyama, Japan; 2 Department of Otorhinolaryngology, Graduate School of Medicine and Pharmaceutical Sciences, University of Toyama, Toyama, Japan; 3 Department of Systems Neuroscience, School of Medicine, Fukushima Medical University, Fukushima, Japan; 4 Faculty of Health Sciences, Mejiro University, Saitama, Japan; University of Oxford, United Kingdom

## Abstract

It has been demonstrated in the rodent hippocampus that rhythmic slow activity (theta) predominantly occurs during rapid eye movement (REM) sleep, while sharp waves and associated ripples occur mainly during non-REM sleep. However, evidence is lacking for correlates of sleep stages with electroencephalogram (EEG) in the hippocampus of monkeys. In the present study, we recorded hippocampal EEG from the dentate gyrus in monkeys overnight under conditions of polysomnographical monitoring. As result, the hippocampal EEG changed in a manner similar to that of the surface EEG: during wakefulness, the hippocampal EEG showed fast, desynchronized waves, which were partly replaced with slower waves of intermediate amplitudes during the shallow stages of non-REM sleep. During the deep stages of non-REM sleep, continuous, slower oscillations (0.5–8 Hz) with high amplitudes were predominant. During REM sleep, the hippocampal EEG again showed fast, desynchronized waves similar to those found during wakefulness. These results indicate that in the monkey, hippocampal rhythmic slow activity rarely occurs during REM sleep, which is in clear contrast to that of rodents. In addition, the increase in the slower oscillations of hippocampal EEG during non-REM sleep, which resembled that of the surface EEG, may at least partly reflect cortical inputs to the dentate gyrus during this behavioral state.

## Introduction

The hippocampus is important for certain types of learning and memory [1]–[6]. There is a growing body of evidence for the contribution of sleep to memory function (for review, see [7]–[11]). As regards the relationship between sleep and hippocampal electroencephalogram (EEG), it has been demonstrated in the hippocampus of rodents that rhythmic slow activity (so-called “theta oscillation”) occurs predominantly during rapid eye movement (REM) sleep, while sharp waves and associated high-frequency (200 Hz) oscillations (“ripples”) intermittently occur during non-REM (slow-wave) sleep [12]–[17]. Although rhythmic slow activity in the hippocampus has different behavioral correlates in different animal species in an awake condition, REM sleep is the behavioral state under which rhythmic slow activity is invariably observed across many nonprimate animals [13], [14].

Whereas there is abundant evidence of the relationship between sleep and hippocampal EEG in rodents, the literature on this issue is very limited in primates, including humans. As for studies using macaque monkeys, to the best of our knowledge, there have been only two reports describing the relationship between the hippocampal EEG and natural sleep in macaque monkeys [18], [19], and in those studies, sleep stage was not classified. Several studies have dealt with the sleep-stage correlates of hippocampal EEG recorded in patients suffering from temporal lobe epilepsy (e.g., [20]–[25]). The findings from these studies are relatively inconsistent. Sources of controversy may have been various differences between experimental techniques and conditions used in the respective studies. Therefore, we think that it is of value to investigate sleep-stage correlates of hippocampal EEG under controlled conditions using monkeys as experimental animals. One of the major barriers to performing investigation of this kind has been the precise positioning of recording electrodes in the deep telencephalic structure (hippocampus) of the primate brain. We have recently resolved these issues by developing a method of recording evoked local field potentials (LFPs) in the hippocampus of behaving monkeys [26]; this technique allowed us to precisely implant recording electrodes in the monkey hippocampus at the subfield or cytoarchitectonic-layer level under conditions of electrophysiological monitoring. In the present study, we implanted recording electrodes in the dentate gyrus of monkeys using this technique, and recorded hippocampal EEG while the monkeys were asleep.

## Materials and Methods

### Subjects

Data were acquired from 2 male monkeys (Macaca fuscata; Monkey-A, 11.0 kg and Monkey-B, 5.0 kg at the beginning of the experiment). The animals were treated in strict compliance with the Animal Care and Use Committee of the University of Toyama, and with the NIH Guide for the Care and Use of Laboratory Animals. The protocol was approved by the University of Toyama's ethics committee for Animal Care and Use (Permit #: S-2009 MED-2). All studies were performed in accordance with the recommendations of the Weatherall report, “The use of non-human primates in research”. The animals were housed individually in a large room for the care of macaque monkeys in the Life Research Center of the University of Toyama, along with several other monkeys permitting rich visual, olfactory and auditory interactions. Water was available *ad libitum*, and a standard commercially formulated nonhuman primate diet (Monkey Bit, Nosan Co., Yokohama, Japan) was provided twice daily (50–80 g/meal) and supplemented daily with fresh fruit. Some toys (such as balls, small stuffed animals, etc.) were given to the monkey as part of the environmental enrichment approach. Regular care and monitoring, balanced nutrition and environmental enrichment were provided by the staffs of this center. All surgery was performed under sodium pentobarbital anesthesia, and all efforts were made to minimize suffering.

### Acclimatization to the recording environment

A cage (80×80×80 cm) with water available *ad libitum* was placed in a temperature-regulated room dedicated for electrophysiological recording. A near-infrared CCD camera equipped with infrared LED illumination was set in front of the cage to monitor the monkey in darkness. To acclimatize the monkey to the recording environment, the monkey was trained to stay in the cage for over night. The monkey was carried to the experimental room and was placed in the cage at approximately 19:00. An animal collar was placed on the monkey, and a leash (flexible metal wire) was connected to the collar. All illumination of the room was turned off at ca. 20:00. Light was turned on automatically using a timer at 6:00 the next morning. The monkey was returned to the home cage between 8:00 and 9:00. This training was repeated twice per week for one month.

### Surgical attachment of cranioplastic cap

The monkey was anesthetized with pentobarbital sodium (35 mg/kg, i.m.) and mounted in the stereotaxic device with the head adjusted in standard stereotaxic planes according to the conventional procedure [26]–[28]. All surgical operations were performed under aseptic conditions. A cranioplastic cap (a head-restraining device) was surgically attached to the skull of the monkey. This cap made it possible to fix the monkey's head in a stereotaxic device painlessly and thus permitted repeated, stereotaxic manipulations for a period of months. The cranioplastic cap was composed of a rectangular epoxy frame, titanium screws, and dental acrylic. The titanium screws were driven firmly into the skull. We adopted 99.9%-fineness titanium screws to reduce artifacts on magnetic resonance (MR) imaging. The epoxy frame was anchored to these screws by dental acrylic, with the horizontal and vertical planes parallel to the orbitomeatal and sagittal planes, respectively. A small titanium screw was attached to the surface of the cap with its tip pointing upward, and the coordinates of the tip from stereotaxic zero (the middle point of the interaural line) were measured. This screw served as the stereotaxic reference in subsequent experimental sessions. To prevent infection, an antibiotic was administered systemically before and after surgery (orbifloxacin, 5 mg/kg, i.m.). To reduce pain, a nonsteroidal anti-inflammatory drug was administered once or twice a day after surgery for a few days (flunixin meglumine, 1 mg/kg, i.m.). The monkey was allowed to recover for more than 2 weeks before implantation of the electrodes as described below.

### Implantation of hippocampal electrodes

**Figure 1:**
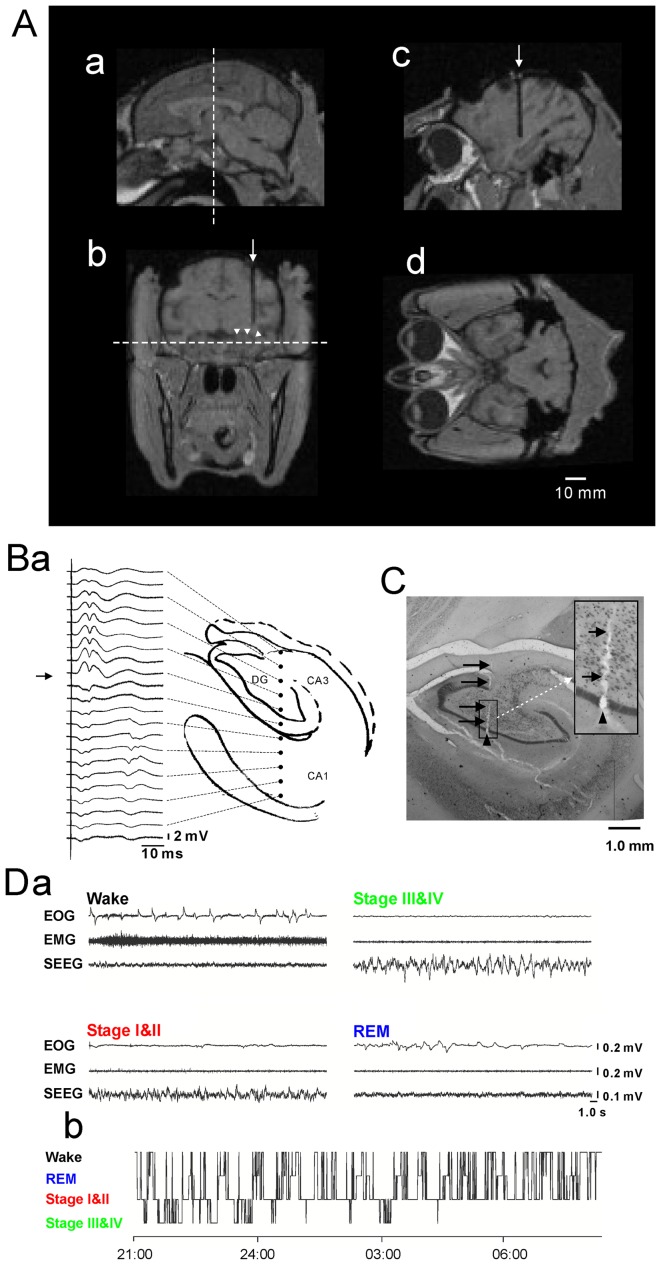
Localization of target brain areas and determination of behavioral states. **A**: Magnetic resonance (MR) images of the monkey brain. **a**: Sagittal image at the midline of the monkey brain. **b**: Coronal image at the level shown by the dashed line in **a**. An arrow indicates a translucent part corresponding to a tungsten marker rod. Arrowheads indicate the approximate border between the hippocampus and the lateral ventricle. **c**: Sagittal image at the level shown by the arrow (i.e., the level of tungsten marker rod) in **b**. **d**: Horizontal image at the level shown by the dashed line in **b**. Horizontal bar at bottom right, calibration scale (10 mm). **B**: Depth profiles of evoked local field potentials. Note that a reversal of local field potential appears at the molecular layer of the dentate gyrus (DG). **C**: an example of histologically identified marking lesion. In this case, the tip of the marking electrode was placed in the dentate granular cell layer under the guide of electrophysiological monitoring (evoked local field potentials and unit activities). Arrows indicate the track of the electrode; an arrowhead indicates the lesion produced by DC current passed through the electrode. **D**: Behavioral states (awake or sleep stages) of the monkey. **a**: Typical examples of polysomnographic recordings. Each example represents recordings while the monkey was awake (Wake), in non-REM sleep stages I and II (Stage I & II), in non-REM sleep stages III and IV (Stage III & IV), or in REM sleep (REM). EOG: electrooculogram, EMG: electromyogram, sEEG: surface electroencephalogram. **b**: A typical example of hypnograms. Overnight changes in sleep stages are depicted.

Concentric electrodes were used for stimulation and recording. These electrodes consisted of an enamel-coated stainless-steel wire (0.1 mm in diameter) encased in a polyurethane-coated stainless-steel cannula (27 G) with an appropriate tip separation (about 2 mm for the stimulation electrode and 5 mm for the recording electrode). The monkey was re-anesthetized with pentobarbital sodium (35 mg/kg, i.m.) and mounted in the stereotaxic device. To form a ground connection, a small stainless-steel screw was driven into the dental acrylic and underlying skull near the midline. A small hole was drilled through the cap and the underlying skull over the target areas, the coordinates of which were determined based on the MR images (Fig. 1A) and X-ray photography performed in advance (technical details for the MR imaging were described in our previous paper [29]) and also based on the brain map [30]. The dura mater was exposed, and small incisions were made into it. The stimulation and recording electrodes were advanced into the brain through the incision, with care taken to aim at positions close to the expected coordinates of the perforant pathway and the dentate gyrus, respectively. The stimulation electrode was connected to a stimulator (SEN-7203, Nihon Kohden) through an isolation unit; the recording electrode was connected to a main amplifier (Lynx-8, Neuralynx) through a high-input impedance preamplifier. A microcomputer equipped with a multifunction board (DT3010, Data Translation) gave rise to a trigger signal (TTL level) to the stimulator every 10 s; the stimulator output a single positive square pulse of 0.2-ms duration (100–200 µA) synchronized to the trigger signal. The output of the main amplifier was monitored on a storage oscilloscope. Both the stimulation and recording electrodes were lowered into the target areas. When evoked LFPs of small amplitude were observed, the stimulation electrode was further advanced while the recording electrode remained at the same depth, in order to determine the optimal depth of the stimulation electrode. Then, the recording electrode was advanced ventrally in 0.1- or 0.2-mm steps and evoked LFPs were recorded. This procedure was repeated with shifts in the insertion coordinates of the recording and/or stimulation electrodes to produce depth profiles of LFPs (Fig. 1B). These depth profiles allowed us to place the electrodes precisely at the most suitable coordinates of the perforant pathway for stimulation, and the hilar region of the dentate gyrus for recording (for details, see [26]). These electrodes were then fixed to the cranioplastic cap using dental acrylic together with lead wires and a small socket.

For Monkey-B, after removal of the concentric recording electrode, we implanted a multiwire electrode for the simultaneous recording of hippocampal field potentials in 4 experimental sessions (Table 1). The multiwire electrode consisted of 7 enamel coated-stainless wires (0.1 mm in diameter); recording contacts were spaced 0.2 mm apart. This electrode was attached to a micro-drive platform. A stainless-steel guide tube coated with polyurethane was inserted just above the dentate gyrus and was fixed to the cranioplastic cap using dental acrylic. This guide tube also served as the reference electrode to the multiwire electrode. The multiwire electrode was then implanted in the target brain region in almost the same manner as was used for the implantation of the concentric recording electrode described above.

**Table 1:** Data sets for recording in the dentate gyrus.

	Sessions			Segments		
	Effective	Ineffective	Total	Effective	Artifacts	Total
Monkey-A	5	3	8	6523	52	6575
Monkey-B	3	1	4	3505	32	3537

### Implantation of surface EEG, EOG, and EMG electrodes

At the same time as the implantation of hippocampal electrodes described above, electrodes of surface EEG, electrooculogram (EOG), and electromyogram (EMG) were also implanted in the monkeys. Briefly, for surface EEG recording, two small stainless-steel screws with a lead wire were driven into the dental acrylic and underlying skull of the frontal pair of the recording sites (corresponding to the electrode placement of F7 and F8 in the international 10/20 system). For EOG recording, a pair of teflon-coated stainless-steel wires (0.1 mm in diameter) was implanted under the skin with the tips positioned at the right and left lateral canthi. For EMG recording, another pair of the teflon-coated stainless-steel wires was implanted with the tips positioned in the dorsal neck muscles (erector muscles of spine). These wires were passed under the skin toward the head; a connecter pin was soldered to the other end of each wire, and this pin was fixed to the cranioplastic cap using dental acrylic.

### Recording procedure and data acquisition

After one week of recovery from the electrode implantation, overnight recording experiments were initiated in the sleeping monkey. Recordings were taken once or twice per week for several months. The monkey was placed between 19:00–21:00 in the same cage as had been used for the acclimatization, and a recording cable (ca. 5 m long) was attached to the connector that had previously been fixed to the cranioplastic cap. The monkey was only loosely tied by the collar and leash, and could move freely in the cage. The near-infrared CCD camera was placed in front of the cage. Each electrophysiological signal was fed to the amplifiers. Signals of hippocampal EEG, surface EEG, EOG and EMG were amplified 2,500, 5,000, 5,000, and 5,000 times, respectively, and were filtered with band-passes of 0.1–325, 0.1–325, 0.1–100, and 10–9,000 Hz, respectively. After it had been confirmed that each electrophysiological signal was appropriately recorded, all room lights were turned off. The lights were then automatically turned on at 6:00 the following morning.

In Monkey-A, hippocampal EEG was recorded in the hilar region of the dentate gyrus for several nights via the concentric recording electrode (Table 1). In Monkey-B, the hippocampal EEG was simultaneously measured at 7 depths using the multiwire electrode; under the guidance of the evoked LFPs, the electrode was advanced ventrally such that the shortest and longest wires appeared to be positioned at the hilar region of the dentate gyrus and the upper portion of the CA1, respectively, crossing the granule cell layer (i.e., the reversal of evoked LFPs was observed between middle pair of the wires).

The bitmap data from the near-infrared CCD camera (1 frame = 320×240 pixels) were acquired by a frame grabber board (DT3152, Data Translation) in the microcomputer at a rate of 30 frames/s; every 30th image was stored on the hard disk. The computer was connected to the internet such that the behavioral state of the monkey could be monitored using any computer equipped with the relevant remote desktop application and internet access. The hippocampal EEG, surface EEG, EOG, and EMG data were acquired at 1,000 Hz via the multi-function board (DT3010, Data Translation) in the microcomputer; these data were stored on the hard disk every 32.768 s ( = 1 segment).

### Data analysis

Because the sleep architecture of monkeys is known to resemble that of humans [31], polysomnographic data in each segment were visually scored using criteria based on the standards for human sleep staging established by Rechtschaffen and Kales [32]. In addition, we scored an epoch as an “artifact” when it consisted of >50% noise and obscured the pattern. The sleep efficiency index (the ratio of total sleep time to total time of the dark period from lights-off in the evening to lights-on the next morning) was calculated based on these polysomnographic and bitmap data.

For the analysis of the hippocampal EEG data, we used results acquired from the hilar region of the dentate gyrus of Monkey-A recorded through the concentric electrode (Table 1). In addition, as regards the findings recorded in the Monkey-B using the multiwire electrode, we calculated “differential data”, by subtracting data recorded through a wire above the granular cell layer from those below the layer.

We carried out spectral analyses of these EEG data, as recorded both through the concentric electrode and the multiwire electrode; Hamming window (32,768 points) was used in 32,768-point Fast Fourier Transform (FFT) to minimize spectral leakage. The full-spectrum amplitude data in each session was first averaged across segments of the same behavioral state. These averaged data were then normalized relative to the peak amplitude appearing in the slow wave frequency (0.5–1.2 Hz) during wakefulness. Finally, these data were averaged across different sessions and different monkeys. The spectral amplitudes calculated by FFT were further averaged in the delta (1.0–4.0 Hz), theta (4.0–8.0 Hz), alpha (8.0–12.0 Hz), beta (12.0–20.0 Hz), and gamma (20.0–80.0 Hz) frequency ranges for statistical purposes.

To identify transient slow (theta and delta) oscillatory events across behavioral states, we used a modification of the technique developed by Caplan et al. [33]. This method enabled us to identify epochs of hippocampal EEG signals that exhibited high-power rhythmic activity at a particular frequency and that lasted more than a few cycles. Briefly, an oscillatory episode at a particular frequency, *f* *, was defined as an epoch longer than a duration threshold *D*_T_ (in number of cycles), during which the power at frequency *f* * exceeded a power threshold, *P*_T_. We first reduced the number of data points of the recorded signal by one-tenth in order to save calculation time. Then, these reduced data were transformed by wavelet analysis (mother wavelet, Morlet; window cycle, 6; Wavelet ToolBox, MATLAB, MathWorks) at 25 logarithmically spaced frequencies in the range of 1–64 Hz. This yielded wavelet power as a function of time (every 10 ms) at each frequency of interest. To eliminate possible edge effects, data on the first 1 s and the last 1 s in each data segment were discarded. Then, the *P_T_* in each frequency was determined by selecting the largest wavelet power in each segment and averaging these values across all segments in one recording session. *D*_T_ at frequency *f* * was set to three cycles (i.e., *f* */3) to eliminate artifacts and physiological signatures that were nonrhythmic. In the present study, the number of rhythmic episodes was counted for the theta and delta ranges.

In the analysis of hippocampal EEG, we calculated the averages and SEMs in four behavioral states of the monkey: wakefulness, non-REM sleep stages I and II (Stage I & II), non-REM sleep stages III and IV (Stage III & IV), and REM sleep. One-way analysis of variance (ANOVA) tests were conducted on the data from the spectral analysis (factor: behavioral state); the ANOVA was followed by the Bonferroni post-hoc test. We used the Chi-square test to detect significant differences in counts in the transient slow oscillatory events (wavelet analysis) between behavioral states. For these statistical tests, the level of significance was set at *P*<0.05.

### Histology

At the end of the experiments, one animal (Monkey-A) was given an overdose of pentobarbital sodium (80 mg/kg, i.m.). A marking electrode was stereotaxically inserted into the brain and positioned at several coordinates in and around the hippocampal EEG recording sites, and 50 µA-DC (negative) current was passed through it for 30 s. The animal was then perfused with 0.9% saline containing heparin followed by 10% buffered formalin. The brain was removed and fixed in the same solution, and placed in 20% sucrose and 10% formalin solution overnight before section. The brain was cut into 50-µm-thick sections on a freezing microtome (coronal section) and stained with cresyl violet to verify the location of the marking lesions (Fig. 1C). The other animal (Monkey-B) is still active and participated in a different experiment from the present study.

## Results

### Data sets of recording experiments

In the present study, we performed a total of 12 recording sessions from the 2 monkeys (Monkey-A: 8 sessions, Monkey-B: 4 sessions). Of these, data from 4 sessions were dropped (3 sessions from Monkey-A and 1 session from Monkey-B) because of troubles in the recording devices or animal conditions such as hyposomnia for unknown reasons (i.e., more than half of the segments recorded were classified as waking state without segments of deep non-REM sleep). There were a total of 6,575 data segments ( = 215,449.6 s) in the 5 effective sessions recorded in Monkey-A with an average of 1,315 segments/session (ranging from 1,171–1,434 segments/session); there were a total of 3,537 data segments ( = 115,900.4 s) in the 3 effective sessions recorded in Monkey-B with an average of 1179.0 segments/session (ranging from 1,054–1,276 segments/session) (Table 1). The data of segments with substantial artifacts were discarded from the subsequent analysis. Judging from the video images recorded by the near infra-red CCD camera, some monkey's behaviors (such as pulling or biting the recording cable) were the main causes of electrical artifacts in EEG signals, although such behaviors were not very often (the average percentage of data per EEG recording session that was deemed artifact was 0.83%).

### Sleep-stage analysis

The polysomnographs acquired in the monkey revealed characteristic changes (Fig. 1Da) that closely resembled those observed in humans. In a state of wakefulness (Wake), a rapid deflection with high-amplitude positive or negative polarity often appeared in the EOG, reflecting large and active eye movements to the right or left; moreover, a high level of activity was observed in the EMG, reflecting a high tone of antigravity muscles (the erector muscles of cervical spine) in a state of wakefulness. In this state, the surface EEG exhibited fast, desynchronized waves. In the early stages of non-REM sleep (Stage I & II), EOG and EMG activity decreased substantially, although small deflections sometimes appeared in the EOG. The surface EEG showed slower oscillations of intermediate amplitude and mixed frequency, with intermittent high-amplitude sharp waves. Sleep spindle-like waves and K complex-like waves were occasionally observed in the stage II of non-REM sleep (Fig. S1). In the late stages of non-REM sleep (Stage III & IV), EOG and EMG activity further decreased and the surface EEG showed continuous, high-amplitude slow waves. In REM sleep (REM), frequent deflections appeared again in the EOG, reflecting rapid eye movements, while EMG activity remained at its lowest level. In this state, surface EEG resembled the fast, desynchronized waves found during wakefulness.

In the present study, the monkeys slept most often in a sitting position (but sometimes in a prone position) for 7–8 hours per night (470.3±20.8 min). The total time of the dark period from light-off in the evening to light-on next morning was 10–11 hours and sleep efficiency index was 73.3%. For the distribution of sleep stages per night, Stage I & II, Stage III & IV, and REM sleep occupied 68.2%, 11.4%, and 20.4%, respectively, of the time spent sleeping. Figure 1Db shows an example of the time course of sleep stages. Slow-wave sleep (Stage III & IV) frequently appeared in the early period of one night of sleep, most of which appeared in the early half of the night of sleeping (early half, 85.8%; late half, 14.2%). Non-REM sleep in the late half of the sleep period consisted primarily of stages I and II; REM also increased in this half of the sleep period. The monkey woke up frequently; this was true, even in the early half of one night of sleep, which was consistent with previous studies reporting that monkeys have less consolidated sleep and a shorter duration for each sleep episode compared with humans [31], [34].

### Hippocampal EEG and spectrum analysis

**Figure 2:**
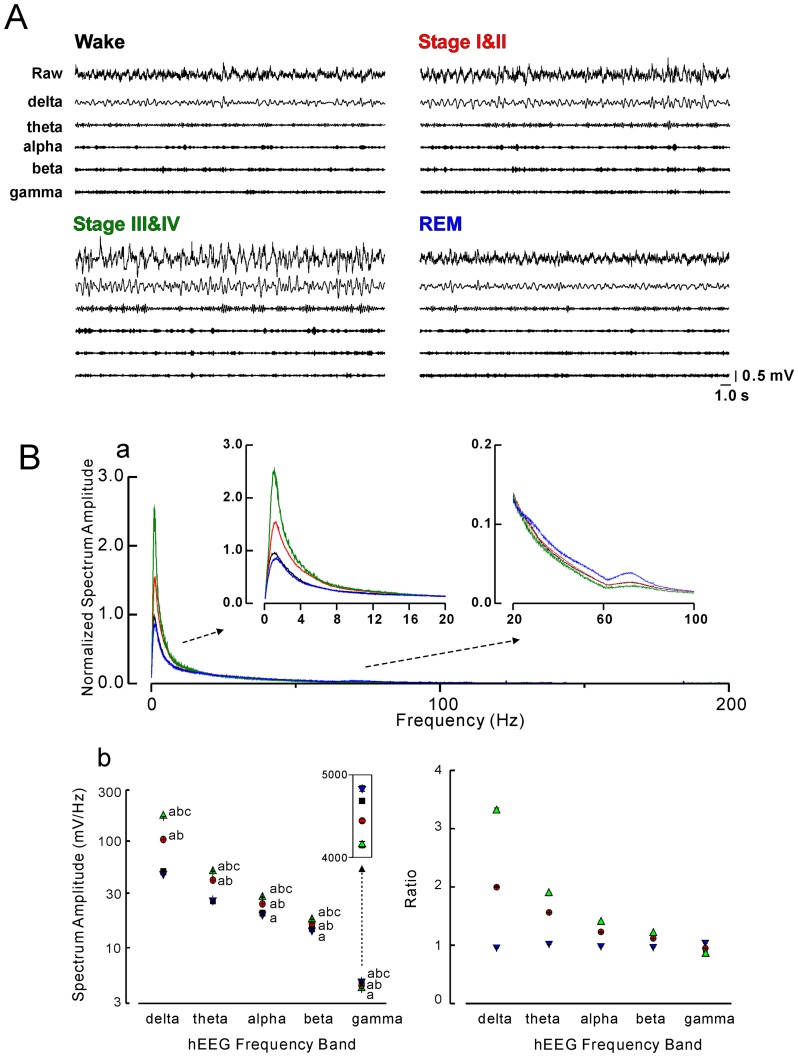
Relationship between hippocampal electroencephalogram (hEEG) and behavioral states. **A**: Typical examples of raw and band (delta, theta, alpha, beta or gamma) pass filtered hEEG recorded from the dentate gyrus during the four behavioral states. **B**: Sleep-stage-dependent changes in the electroencephalographic spectrum recorded in the dentate gyrus during the four behavioral states. **a**: Normalized full-spectral amplitude of hEEG. **b**: Absolute (left panel) and normalized (right panel) spectral amplitudes in each frequency band (from delta to gamma) of hEEG. Black, red, green, and blue lines or symbols represent data recorded during wakefulness, Stage I & II, Stage III & IV, and REM sleep, respectively. Letters ‘a’, ‘b’ and ‘c’ next to each symbol represent significant differences (Bonferroni multiple comparison, *P*<0.05) from Wake, REM and Stage I & II, respectively.

As shown in Fig. 2A, the EEG recorded in the dentate gyrus changed in a manner similar to that of the surface EEG: during wakefulness, the EEG showed characteristic fast, desynchronized waves, which were partly replaced with slower waves of intermediate amplitudes during Stage I & II. During Stage III & IV, continuous, slow waves with high amplitudes were predominant. In REM, the hippocampal EEG also resembled that found during wakefulness. The spectrum analysis confirmed these findings. Figure 2Ba shows the normalized full spectrum amplitude of EEG recorded in the dentate gyrus during the four behavioral states. The patterns of spectral amplitudes in the lower to middle frequency bands (delta to beta) were similar between Wakefulness and REM except for a slight decrease in the lowermost frequency (0.5–1.5 Hz) during REM (Fig. 2Ba, and its left inset). The spectral amplitude increased in the low-frequency range as the monkey descended into deeper, non-REM sleep from Stage I & II to Stage III & IV (Fig. 2Ba). These sleep-stage-dependent changes were diminished at higher frequencies and were reversed at the highest frequency (gamma). In particular, the spectrum amplitude of this band was increased during REM (blue line in Fig. 2Ba right inset). To perform statistical analysis, we binned the spectrum amplitude data into 5 frequency bands (delta, theta, alpha, beta and gamma) as described in **Materials and Methods**. The results of this analysis are shown in the left panel of Fig. 2Bb. One-way ANOVA tests revealed significant main effects of behavioral states in each frequency band [for delta, F(3,10024) = 4694.73; for theta, F(3,10024) = 2251.23; for alpha, F(3,10024) = 534.23; for beta, F(3,10024) = 490.26; for gamma, F(3,10024) = 74.87; all *Ps*<0.05]. The multiple comparison test (Bonferroni) confirmed the effect of behavioral state on the spectrum amplitudes in each frequency band mentioned above. This comparison across spectrum bands might have been biased by the absolute spectrum amplitude since, by nature, slower-frequency bands tend to have larger spectrum amplitudes than faster-frequency bands. To remove this bias, we also compared normalized spectral amplitudes, which were calculated as the ratio of spectral amplitude for each sleep stage to that for wakefulness (Fig. 2Bb, right panel). This comparison revealed qualitatively the same results.

**Figure 3:**
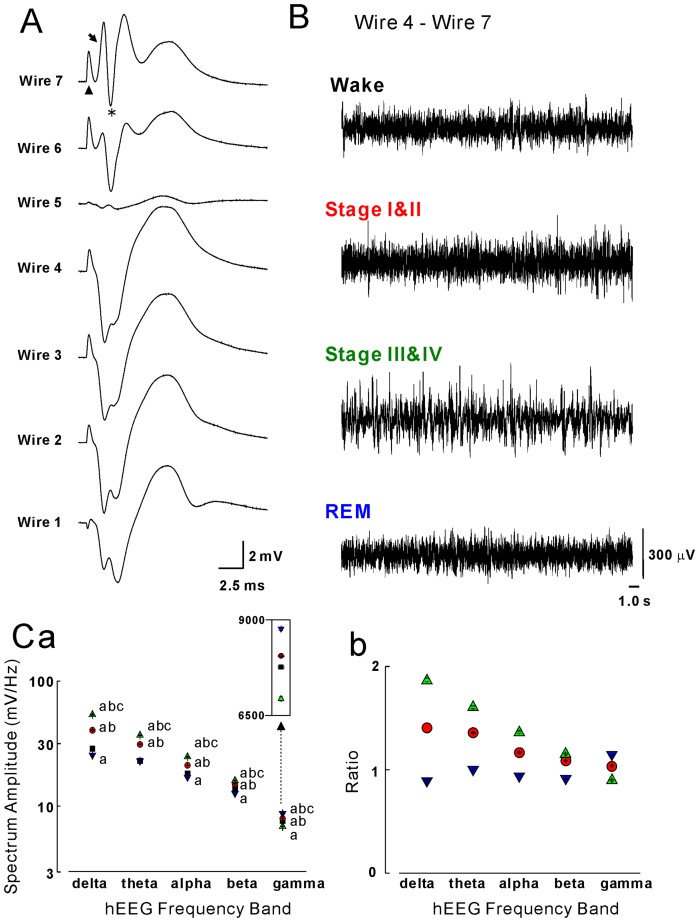
Sleep-stage-dependent changes in differential recordings of the hippocampal electroencephalogram (hEEG) during the four behavioral states. **A**: Depth profile of evoked local field potentials recorded through a multiwire electrode. Upright triangle, arrow, and asterisk indicate the stimulation artifact, the rising phase of field excitatory postsynaptic potential, and the population spike, respectively. **B**: Typical examples of “differential” (Wire 4-Wire 7) data on hEEG recorded from the dentate gyrus during the four behavioral states. **C**: Absolute (**a**) and normalized (**b**) spectral amplitudes of hEEG recorded from the dentate gyrus, in the delta, theta, alpha, beta, and gamma frequency bands, based on the data of the “differential” recordings. Other descriptions are the same as for **Fig. 2**.

The abovementioned sleep-stage correlates of the hippocampal EEG could be ascribed to effects on signals recorded via the reference electrode (the outer cannula of the concentric electrode) or far-field potentials possibly produced in adjacent cortices; namely, changes in the EEG within the dentate gyrus would not account for these correlates. To rule out or minimize the possibility of confounding effects, we also analyzed “differential” data (see **Materials and Methods**). Figure 3 shows the results from this analysis. The depth profile of evoked LFPs shows a potential reversal at or around Wire 5 (Fig. 3A). Therefore, the tips of Wire 4 and Wire 7 were thought to be located in the outer molecular layer and the hilar region, respectively, of the dentate gyrus. The data from these electrodes were used for the calculation of differential data. A pattern of sleep-stage dependence similar to that acquired through the concentric electrode appeared in this analysis (Fig. 3B). This pattern was again quantitatively confirmed by spectrum analysis (Fig. 3C, a and b). These results demonstrated that sleep-stage-dependent changes in hippocampal EEG occur within the dentate gyrus.

### Wavelet analysis of hippocampal EEG

**Figure 4:**
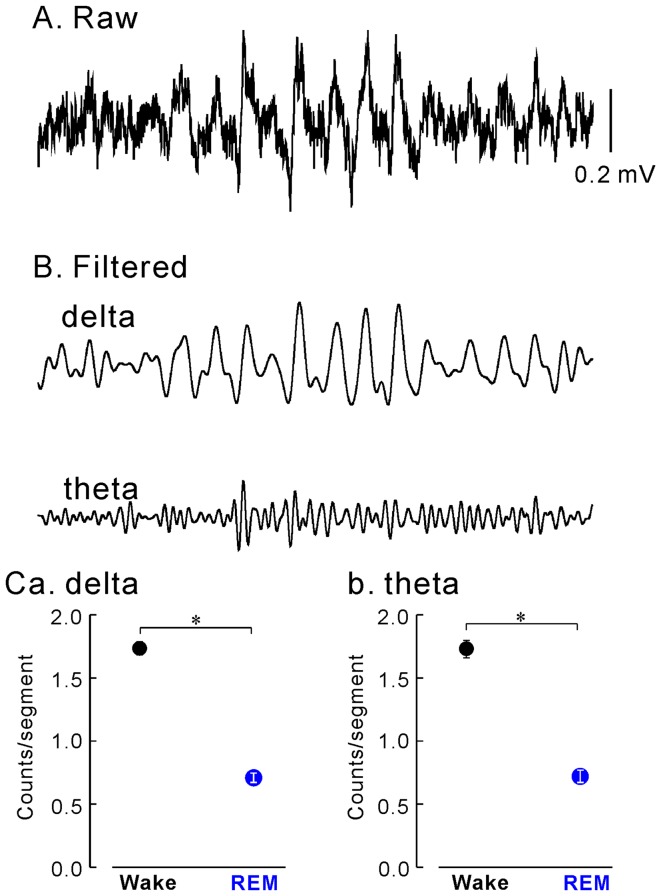
Transient slower oscillatory (theta and delta) events. **A and B**: Typical examples of raw (**A**) and band-pass filtered (**B**, delta or theta band) hippocampal encephalograms of transient slow oscillatory events recorded in the dentate gyrus. **C**: Comparisons of the number of transient slow oscillatory events in delta (**a**) and theta (**b**) bands between wakefulness and REM sleep. Asterisks (*), *P*<0.05.

As described above, in the present study we did not observe continuous rhythmic slow activity in the dentate gyrus while the monkey was in REM sleep. However, this does not mean that transient slow (delta and theta) oscillatory events increase during REM sleep, which was reported in the human hippocampus by Cantero et al. [23]. To test whether the frequency of transient slow oscillatory events increases during REM sleep, we counted these events using a wavelet analysis (for the details of this analysis, see *Wavelet analysis of the hippocampal EEG* in **Materials and Methods**). Typical examples of the transient slow oscillatory events are shown in Fig. 4, A and B. The number of transient slow oscillatory events in the dentate gyrus during REM sleep was significantly decreased, both in the delta and theta ranges (Fig. 4C, a and b, respectively), as compared to that associated with wakefulness [a total of 8 sessions (10,028 segments) recorded from the 2 monkeys; Chi square test, *P*<0.05].

## Discussion

### Relations of monkey hippocampal EEG with rhythmic slow activity in rodents

Although hippocampal rhythmic slow activity has different behavioral correlates in different animal species, REM sleep is the behavioral state under which rhythmic slow activity is invariably observed across many nonprimate animals [13], [14]. However, in the present study, we did not observe clear, continuous rhythmic slow activity during REM sleep. This presents a clear contrast between animal species. Also, previous studies investigating hippocampal neural activity in the naturally sleeping monkey did not find rhythmic slow activity during REM sleep (e.g., [18], [35]), consistent with the results of the present study. This difference between animal species may also be supported by evidence showing that, in a state of wakefulness, continuous rhythmic slow activity appears in rodents when the animal performs a category of behaviors such as exploring, jumping, and rearing [12], [36], whereas rhythmic slow activity occurs only very transiently, in monkeys performing these categories of behaviors ([37]; Tamura et al., unpublished observation). It has also been reported that, in other animal species such as bats, rhythmic slow activity rarely appears in the hippocampus or in the medial entorhinal cortex during either sleeping or waking states; furthermore, even if rhythmic slow activity appears during a particular type of behavior (echolocation), it is not continuous, but occurs in short intermittent bouts [38], [39].

The absence of clear continuous rhythmic slow activity in the monkey dentate gyrus during REM sleep was not thought to be due to the differential recording configuration of the concentric electrode used in the present study. It is known that in the rat, the amplitude of theta oscillation recorded in the hilar region of the dentate gyrus is high in the portion from the center of this region to the crest of the granular cell layer (i.e., near the electric dipole generated by granular cell assembly), and it decreases as the recording site is away from the dipole toward the CA3 region (e.g., [40]). In the present study, the tip of the electrode inner wire was located around the center of the hilar region, and that of the outer (concentric) cannula, separated by ca. 5 mm from the tip of the inner wire, was located above and outside of the hilar region (probably in the ventral part of the thalamus near the lateral ventricle). Therefore, we believe that if there had been rhythmic slow activity with substantial amplitudes generated in the dentate gyrus during REM sleep, it could have been detected through the concentric electrode. In addition, we recorded the hippocampal EEG using the multiwire electrode and calculated the “differential data” using signals recorded in the hilar region and those recorded below the granular cell layer near the hippocampal fissure. It is known that in the rat, there is a large phase shift between rhythmic slow activity recorded in the hilar region and that recorded around the hippocampal fissure [41]. Therefore, the calculation of differential data must have optimized the detection probability of rhythmic slow activity generated in the dentate gyrus.

Rather, slower oscillation (delta and theta) in the dentate gyrus was more obvious during the deep stages of non-REM sleep, in which slow waves (<1 Hz) and delta waves occurred predominantly in the neocortical areas. We had suspected that the slower oscillation recorded in the dentate gyrus via a conventional concentric recording electrode might have been volume-conducted EEG oscillation (i.e., far-field potentials) generated in the neocortical areas. Therefore, to minimize this effect, we used the differential data to cancel out any common inputs of far-field potentials. Nevertheless, we obtained basically the same results from these data, which provided compelling evidence that the slower oscillation recorded in the dentate gyrus during the deep stages of non-REM sleep was due not to far-field effects from the neocortical areas (nor potential changes in the region of the reference electrode); rather, this oscillation occurred in the dentate gyrus *per se*. Because of the large differences in behavioral correlates, we speculate that the slower oscillation is not the primate homolog of rhythmic slow activity in rodents. This oscillation could reflect oscillatory inputs from cortical regions (e.g., the entorhinal cortex) during slow wave sleep. It has been found that synchronous neocortical patterns associated with slow waves invade and affect the rodent hippocampus [42]–[46]. Specifically, Isomura et al. conducted a current source density analysis on the hippocampal field potentials triggered by the down-up states transitions in sleeping rats, and found that a current sink appears in the dentate molecular layer which receives inputs from entorhinal layer 2 and 3 cells [46]. They also stated that neurons in the rat dentate gyrus are preferentially active during the up state of slow oscillations. These findings indicate that dentate neurons receive rhythmic excitatory synaptic activation from the entorhinal cortex during the up state of slow wave sleep; this could also be the case in the present study. We cannot state this definitively, however, because we did not investigate how the slower oscillation is generated in the monkey hippocampus during non-REM sleep. Further studies are necessary to elucidate the mechanism by which this slower oscillation is generated.

### Sleep correlates of hippocampal EEG in humans

There are series of studies investigating the sleep-stage correlates of the hippocampal EEG in humans. However, the results from these studies are inconsistent, especially as regards rhythmic slow activity and REM sleep. For example, on the one hand, Halgren et al. reported that visual inspection of EEG recorded in the hippocampus of patients suffering from temporal lobe epilepsy failed to reveal rhythmic slow activity during paradoxical sleep [20]. Uchida et al. also found that theta activity was rarely observed in the cortices surrounding the hippocampus during REM sleep (rather, they noticed an increase in beta power during REM sleep) [22], [24]. Furthermore, Moroni et al. recently recorded the intracerebral stereo-EEG directly from the hippocampus and found that the spectrum powers during non-REM sleep were higher than those during REM sleep in a wide frequency range (from 0.5 to 30 Hz), although the extent (power ratio of non-REM/REM) differed in different frequency ranges [25]. On the other hand, Bodizs et al. used mesiotemporal corticography with foramen ovale electrodes in epileptic patients and reported the presence of a low-frequency (from 1.5 to 3 Hz) synchronous rhythmic hippocampal oscillation specific to REM sleep [21]. Cantero et al. reported that transient bursts of theta oscillations appear in the hippocampus and surrounding cortices during REM sleep [23]. The discrepancies between these studies may be due to various differences in such factors as recording techniques, subfields and layers where the signals were recorded, pathological conditions (e.g., epilepsy). In the present study, we precisely implanted recording electrodes in the hilar region of the dentate gyrus of “healthy” monkeys (without epilepsy) under the guide of electrophysiological monitoring. We also used not only conventional concentric electrodes but also multiwire electrodes to rule out or minimize the influence of far-field potentials and voltage changes at the reference point. Using these experimental controls, we found continuous slower oscillation in the monkey hippocampus during deep stages of non-REM sleep, whereas such findings were rare during REM sleep. Furthermore, the occurrence of transient slow oscillatory events was lower during REM sleep than during wakefulness. In this sense, therefore, the present findings were more consistent with the former group of studies.

### Functional significance of sleep-stage-dependent changes in hippocampal EEG

Continuous rhythmic slow activity is commonly observed in the hippocampus of lower mammals [13]. It has been hypothesized that, from the findings in rats, theta oscillation works as an internal clock for the on-line encoding of sensory input during learning and memory while the animal is awake, and for the off-line processing of information during REM sleep [7], [40]. However, in the present study, we did not observe continuous rhythmic slow activity in the monkey hippocampus during REM sleep. Therefore, in the monkey hippocampus, off-line information processing might occur in a manner different from that of the rodent hippocampus.

In contrast to the absence of rhythmic slow activity during REM sleep, slower oscillations clearly appeared in the monkey dentate gyrus during the deep stages of non-REM sleep. As described above, this could reflect oscillatory inputs from cortical regions during slow wave sleep. The interactions (“dialog”) between the hippocampal circuits and neocortical areas during non-REM sleep is thought to be important for memory consolidation [17], [43], [47]–[49]. Therefore, the slower oscillations in the dentate gyrus during slow wave sleep observed in the present study might contribute to the process of memory consolidation, although more compelling evidence from future studies will be needed to confirm this function.

## Supporting Information

Figure S1 **Surface electroencephalogram (EEG) recorded during non-REM sleep stage II.** **A**: Examples of surface EEG traces (10 s) during non-REM sleep stage II. F7 and F8, the recording sites corresponding to the electrode placement of F7 and F8, respectively, in the international 10/20 system. Asterisks (*), K complex-like waves; arrows, sleep spindle-like waves. **B**: Normalized spectral amplitude of surface EEG recorded during non-REM sleep stage II. An arrow indicates a small spectrum peak observed around 16 Hz.
(DOC)
