# Supplementary material for: Sleep-Stage Correlates of Hippocampal Electroencephalogram in Primates
Source: PLoS One. 2013 Dec 30;8(12):e82994. doi: 10.1371/journal.pone.0082994 (PMC3875423; doi:10.1371/journal.pone.0082994)
Supplement: Figure S1 — Surface electroencephalogram (EEG) recorded during non-REM sleep stage II. A: Examples of surface EEG traces (10 s) during non-REM sleep stage II. F7 and F8, the recording sites corresponding to the electrode placement of F7 and F8, respectively, in the international 10/20 system. Asterisks (*), K complex-like waves; arrows, sleep spindle-like waves. B: Normalized spectral amplitude of surface EEG recorded during non-REM sleep stage II. An arrow indicates a small spectrum peak observed around 16 Hz. (DOC) [file pone.0082994.s001.doc]

**
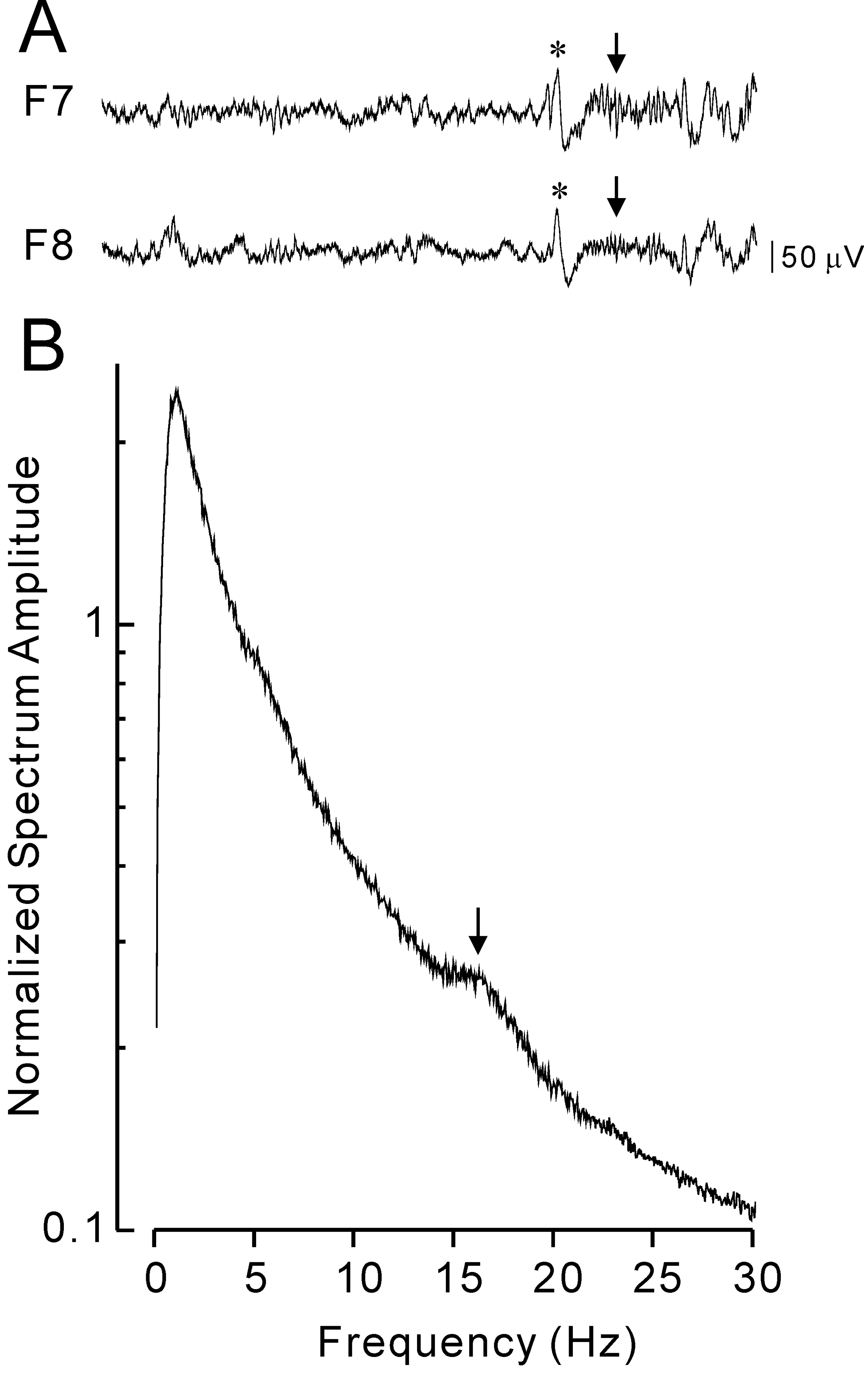
**

**Figure S1. Surface electroencephalogram (EEG) recorded during non-REM sleep stage II.**

**A**: Examples of surface EEG traces (10 s) during non-REM sleep stage II. F7 and F8, the recording sites corresponding to the electrode placement of F7 and F8, respectively, in the international 10/20 system. Asterisks (*), K complex-like waves; arrows, sleep spindle-like waves. **B**: Normalized spectral amplitude of surface EEG recorded during non-REM sleep stage II. An arrow indicates a small spectrum peak observed around 16 Hz.
